# Identifying structural risk factors for overdose following incarceration: a concept mapping study

**DOI:** 10.1186/s40352-024-00265-w

**Published:** 2024-03-12

**Authors:** Samantha K. Nall, Cole Jurecka, Anthony Ammons, Avel Rodriguez, Betsy Craft, Craig Waleed, Daniel Dias, Jessie Henderson, Joshua Boyer, Kristina Yamkovoy, Pallavi Aytha Swathi, Prasad Patil, Forrest Behne, Katherine LeMasters, Lauren Brinkley-Rubinstein, Joshua A. Barocas

**Affiliations:** 1 School of Medicine, University of Colorado, Anschutz Medical Campus, 8th Floor, Academic Office 1 Mailstop B180 12631 E 17th Ave, Aurora, CO 80045 USA; 2 The Ahimsa Collective, Oakland, CA USA; 3 Third City Community Advisory Board, Chapel Hill, NC USA; 4 WORTH CAB (Wellness, Opportunity, Resilience Through Health Community Advisory Board), Aurora, CO USA; 5 Colorado Drug Policy Coalition, Denver, CO USA; 6 Disability Rights North Carolina (DRNC), Raleigh, NC USA; 7 Denver Dream Center, Denver, CO USA; 8 Hopwood and Singhal PLLC, Alexandria, VA USA; 9 Boston University School of Public Health, Boston, MA USA; 10 University of North Carolina at Chapel Hill, Chapel Hill, NC USA; 11 Duke University, Durham, NC USA

**Keywords:** Concept mapping, Community-engaged research, Overdose, Substance use disorder, Incarceration, Qualitative

## Abstract

**Background:**

Currently, there are more than two million people in prisons or jails, with nearly two-thirds meeting the criteria for a substance use disorder. Following these patterns, overdose is the leading cause of death following release from prison and the third leading cause of death during periods of incarceration in jails. Traditional quantitative methods analyzing the factors associated with overdose following incarceration may fail to capture structural and environmental factors present in specific communities. People with lived experiences in the criminal legal system and with substance use disorder hold unique perspectives and must be involved in the research process.

**Objective:**

To identify perceived factors that impact overdose following release from incarceration among people with direct criminal legal involvement and experience with substance use.

**Methods:**

Within a community-engaged approach to research, we used concept mapping to center the perspectives of people with personal experience with the carceral system. The following prompt guided our study: “What do you think are some of the main things that make people who have been in jail or prison more and less likely to overdose?” Individuals participated in three rounds of focus groups, which included brainstorming, sorting and rating, and community interpretation. We used the Concept Systems Inc. platform *groupwisdom* for our analyses and constructed cluster maps.

**Results:**

Eight individuals (ages 33 to 53) from four states participated. The brainstorming process resulted in 83 unique factors that impact overdose. The concept mapping process resulted in five clusters: (1) Community-Based Prevention, (2) Drug Use and Incarceration, (3) Resources for Treatment for Substance Use, (4) Carceral Factors, and (5) Stigma and Structural Barriers.

**Conclusions:**

Our study provides critical insight into community-identified factors associated with overdose following incarceration. These factors should be accounted for during resource planning and decision-making.

**Supplementary Information:**

The online version contains supplementary material available at 10.1186/s40352-024-00265-w.

## Background

Since 1980, the number of incarcerated people in the United States (US) has quadrupled (Ghandnoosh et al., 2023; *Incarceration Statistics*, n.d.; Pfaff, 2015). This drastic increase began in the 1970s when the Nixon-era War on Drugs began in an attempt to be “tough on crime” and limit drug use and distribution (Pfaff, 2015). More than two million people are in jails or prisons in the US, and over 200,000 people are incarcerated at any given time for drug offenses (Ghandnoosh et al., 2023; Pfaff, 2015). People who have been incarcerated – for drug-related reasons or otherwise – are disproportionately affected by substance use disorders (SUDs) and overdose with nearly two-thirds of people in prisons or jails meeting the criteria for a SUD (Bronson, 2017; *Overdose Deaths and Jail Incarceration*, n.d.). In addition to the large proportion of incarcerated people who have a SUD, fatal overdoses are the leading cause of death following release from prison and the third leading cause of death during incarceration in jails (Binswanger et al., 2013; Fiscella et al., 2020; *Overdose Deaths and Jail Incarceration*, n.d.). Specifically, incarcerated individuals are at least 40 to 129 times as likely to die from a drug overdose compared to the general public two weeks following their release (Binswanger et al., 2007; *Overdose Deaths and Jail Incarceration*, n.d.; Ranapurwala et al., 2018). Furthermore, there was a 600% increase in deaths related to alcohol intoxication or drug overdoses in state prisons from 2001 to 2018 and over a 200% increase in county jails (Schwartzapfel & Jenkins, 2021).

Due to the disproportionate prevalence of SUD and risk of overdose that individuals with carceral involvement experience, jails and prisons across the US are beginning to incorporate medications for opioid use disorder (MOUD) and harm reduction strategies (e.g., overdose education, naloxone distribution) for incarcerated individuals and linking them to peer support and treatment post-release. Although the current number of carceral facilities offering these programs is not well documented, many entities advocate for the incorporation of MOUD and harm reduction in prisons and jails (Brinkley-Rubinstein et al., 2017; LAPPA, 2023; Stack et al., 2022; Wenger et al., 2019). However, each state has its own laws and regulations regarding MOUD requirements and harm reduction strategies, which may cause difficulty in achieving uniform results of reducing overdose (Lieberman & Davis, 2020).

Previous work has demonstrated that several factors are associated with increased overdose risk following incarceration, including physiologic loss of tolerance, limited or no access to MOUD or naloxone, and interruption or lack of health care and social support (Brinkley-Rubinstein et al., 2018; Joudrey et al., 2019; *Overdose Deaths and Jail Incarceration*, n.d.). Much of this work is based in quantitative surveys or administrative data records. These traditional methods are necessary, but may fail to capture the complex structural and environmental factors that influence overdose within communities (Cargo & Mercer, 2008; Crawford-Browne & Kaminer, 2012). Moreover, they often do not explicitly include the perspectives of people with lived experience (PWLE), leaving a void in our understanding of what influences overdose from individuals’ perspectives.

People with direct involvement with the criminal legal system hold unique perspectives and insight that can provide crucial information. Community-engaged research (CEnR) that involves people with living or lived experience can rebalance power dynamics by centering on and elevating voices of communities that are often marginalized, informing the design of interventions tailored to specific communities (Swierad & Huang, 2021), and co-creating a knowledge base to have substantial social impacts on the relationship between carceral involvement, substance use, and overdose (Crawford-Browne & Kaminer, 2012; Jull et al., 2017). For example, one study using a CEnR approach found several previously unidentified risk factors (e.g., knowledge of an imminent reincarceration) and several protective factors (e.g., having children, presence of a caseworker when accessing services, positive relationship with a probation officers) for overdose in previously incarcerated individuals (Flam-Ross et al., 2022).

Concept mapping is a CEnR mixed-method approach that uses a single prompt to evoke a conceptual understanding of an issue by having participants map the relationships and interactions between factors (Crawford-Browne & Kaminer, 2012; Kane & Trochim, 2007). Participants then guide the formation of the final concept map while the research team asks clarifying questions regarding themes and discussion points. This approach allows for a “clean slate” regarding conversations about overdose risk, substance use, and incarceration from the perspectives of PWLE. Concept mapping has been employed in several settings, including domestic violence, end-of-life initiatives, and substance use (Crawford-Browne & Kaminer, 2012; Rao et al., 2005; Windsor, 2013). Of the concept mapping studies related to substance use and criminal legal involvement, each focuses on only one of these topics or a different connection (e.g., incarceration and HIV) (Antoniou et al., 2019; Pauly et al., 2022; Urbanoski et al., 2020). It is, however, underutilized in understanding the relationship between criminal legal involvement and overdoses. In this study, we used concept mapping methods to identify factors that people with experience with the criminal legal system perceive as influencing overdose and substance use following incarceration.

## Methods

### Participants and setting

We employed a concept mapping approach in collaboration with the Wellness, Opportunity, Resilience Through Health (WORTH) Program Community Advisory Board (CAB) and the 3rd City Project CAB. These CABs are composed of individuals who have personal experience within the carceral systems and with substance use. WORTH is a program through the University of Colorado School of Medicine that aims to assist with the transition of being released from jail, and to empower individuals to manage their health care needs post-release and while incarcerated (Glasgow, 2023). The WORTH CAB was created to bring those with lived experience together to serve as a key community partner and inform the WORTH program. Similarly, the 3rd City Project CAB was created to guide the project’s research, an initiative meant to improve carceral data transparency relating to health across the US (“Third City Project - CAB Members,” n.d.). Initially, research members (SN, CJ, FB, JB, KL, LBR) reached out to the CAB leaders to share the project idea and report back if members of the CABs were interested in participating. As there was interest from both CABs, we hosted an introductory meeting where the research team explained the objectives and processes of the project and solicited interest. We recruited members from these CABs because of their living and lived experience within the carceral system and substance use, and their unique perspectives.

Participants for this study were 18 or older and had living or lived experience in the US carceral system and with a SUD. Approval for the study was obtained from the Colorado Multiple Institutional Review Board (COMIRB).

### Data collection

We conducted focus groups between October and December 2022 over three virtual sessions. Two members of the research team conducted these discussions, one led the discussion (SN) and the other took notes (CJ). We held an introductory meeting for prospective participants to introduce the purpose of the study and discuss the process of concept mapping through a presentation with a question and answer session. Eight interested individuals recruited from both the WORTH and 3rd City CABs attended the introductory meeting, completed the consent, and participated in the focus group sessions. Sessions 1 through 3 consisted of participants engaging in the concept mapping process. A syllabus with details of the discussions of each session can be found in the Supplemental Appendix. Participants were then compensated ($25 per session) for participating in each session following the introductory meeting. All sessions lasted approximately one hour and were audio-recorded and anonymously transcribed by the research team (SN and CJ). We invited co-authorship to all participants for manuscripts resulting from the project.

#### Brainstorming

During Session 1 (the “brainstorming session”), we asked participants (*N* = 7) to create a list of factors in response to the following focus prompt: “*What do you think are some of the main things that make people who have been in prison or jail more likely and less likely to overdose?*” Participants first considered factors contributing to an increased likelihood of overdose and then factors that may make overdose less likely. We also asked probing questions to facilitate the discussion and prompt the participants to discuss specific factors related to the main question. Following Session 1, the research team compiled a consolidated and refined list of factors to remove duplicates and combine analogous ideas, so that the list of factors contained unique ideas and statements.

#### Sorting and rating

During Session 2 (the “sorting and rating session”), we asked participants to review the final list of factors and ensure that it was accurate and inclusively representative of comments from Session 1 (Cargo & Mercer, 2008; Minkler, 2005). All participants (*N* = 7) from Session 1 were present for Session 2 (*N* = 8), in addition to one participant who was unable to attend Session 1. The participants felt that the factor list was comprehensive and adequately representative of their perspectives and beliefs, and no additional changes needed to be made. We then asked participants to sort the factors into between 2 and 30 piles that they deemed to be similar in the context of the focus prompt and instructed them to name each pile. Each participant had an account in the *groupwisdom* software, and they logged in to complete both the sorting and rating activities. Once factors were sorted, we instructed participants to rate each factor on a 5-point Likert scale relating to (1) how much each factor was related to overdose and (2) how common the factor is within the community. We left the term “community” open to interpretation for each participant because community can mean different things to each person, but participants were informed that we were interested in understanding the perspectives of PWLE.

#### Community interpretation

During Session 3 (“community interpretation session”), we discussed how the sorting and ratings had been generated into concept maps and then asked participants (*N* = 4) to analyze three cluster permutations that the research team presumed most representative of the focus prompt and the participants’ responses. Participants were encouraged to review the names assigned to the clusters and provide feedback (Israel et al., 1998; Minkler, 2005). We alternated between the three maps and explained to participants how each map differed. The participants discussed which version of the concept map they believed to be most representative of their prior discussions, asking questions of the research team about how each map differed. Based on these discussions, the research team created and sent around a presentation that displayed the differences in the factors and clusters for each of the map options. Following this discussion, the participants chose the map that they felt best captured their perceptions.

### Data analysis

For Session 1 (“brainstorming”), the brainstorming discussion was an iterative process where participants were given the prompt, “*What do you think are some of the main things that make people who have been in prison or jail more likely and less likely to overdose?*” and asked to list factors that they believed answered the question. Participants first focused on the “more likely” aspect before moving on to the “less likely” component. Throughout the discourse, participants were asked probing questions by the research team (SN), based on the factors that participants began to list to garner further ideas and statements from the participants. Probing questions included but were not limited to, “*How do you perceive X to influence substance use and overdose*?” and “*Can you elaborate on X regarding your experiences?*” Following Session 1, the research team (SN and CJ) reviewed factors from both brainstorming groups. We compiled a comprehensive list in which we deleted analogous ideas (e.g., stigma and tolerance) and separated concepts encompassing multiple factors. (e.g., “ Increased prevalence of healthcare while incarcerated and bridging that gap after being released”).

For Session 2 (“sorting and rating”), we used the Concept Systems software, *groupwisdom*, to perform our statistical analysis (*The Concept System® Groupwisdom™*, 2021). Based on the *groupwisdom* algorithm, at least 75% of the factors must be sorted for a participant’s data to be included in analysis, which all participants did. First, we organized data into a similarity matrix, visually representing how often participants categorized factors together (“Group Concept Mapping Resource Guide,” 2023). Once the similarity matrix was compiled, we used a multidimensional scaling algorithm to create a point map (“Group Concept Mapping Resource Guide,” 2023). The points on this map represent each of the factors, with distances between points representing how often factors were sorted together by participants (e.g., closer placement indicators frequent similar sorting) (“Group Concept Mapping Resource Guide,” 2023). Each point map is assigned a stress value. The stress value indicates that the two-dimensional solution of the multidimensional analysis fits the data points (goodness of fit). *Groupwisdom* utilizes Ward’s Method, to minimize within-cluster variation, and implement a hierarchical cluster analysis to create a variety of cluster maps (*Distances between Clustering, Hierarchical Clustering*, 2009; “Group Concept Mapping Resource Guide,” 2023). The clusters were created to each contain the factors that participants most frequently sorted together. Each cluster is formed with a different shape. with thinner shapes indicating a higher degree of agreement between individual participant sorting and thicker cluster indicating more dissimilarity. Additionally, larger clusters have more factors within them, and smaller clusters have fewer factors. The research team (SN, CJ, KL, LBR, JB) generated 11 unique cluster maps using *groupwisdom*, and of these, three cluster maps were presented to participants at Session 3 for final selection. The research team analyzed the discussions and the activities (i.e., brainstorming, sorting, and rating) to select the three best map options that were believed to accurately represent the perceptions of the participants. During Session 3, participants were instructed to make their final decision with the following information in mind: (1) a larger cluster is indicative of less agreement between the sorting data, and (2) the proximity of factors on the edges of one cluster to another cluster can provide insight regarding the associations between clusters and their components. For example, factors 51 and 70 as noted in Table 1 in the *Stigma and Structural Barriers* cluster, are in close proximity to factor 11 of the *Resources for Treatment for Substance Use* cluster. Thus, a discussion around each of the cluster maps was held by the participants, with the researcher team answering any questions (SN) about what factors were in which cluster and why. Lastly, the research team (SN and CJ) created a Pattern Match chart and a Go-Zone chart to demonstrate the results from our two rating questions. These visualizations use a bivariate analysis to create a pictorial representation of the relatedness of clusters (Pattern Match) and individual factors (Go-Zone).

**Table 1:** List of numbered factors within each of the five clusters

Community-Based Prevention	Drug Use and Incarceration	Resources for Treatment for Substance Use	Carceral Factors	Stigma and Structural Barriers
1. Substance use counseling	31. Drugs are expensive, so people use more when they have the opportunity	5. Harm reduction communities	9. Harm reduction support for people that get released after-hours	15. Feeling empowered
2. Trauma-informed staff	32. People return back to the same level of drug use after being released	8. People with lived experience as overdose response and peer support	10. Coordinated releases	23. Stigma surrounding possessing Naloxone/Narcan
3. Opportunities to rise above the bare minimum	41. Eliminating stigma from supervising authority	11. Employment opportunities with a living wage	14. Ensuring basic needs are met, especially after release	26. Length of time incarcerated
4. Education on overdose prevention	56. Fentanyl is in everything	12. Adequate healthcare insurance	16. Wellness Recovery Action Plans (WRAP)	27. Arrests can play a role in self-esteem, leading to a higher likelihood of using
6. Other recovery programs besides abstinence-based recovery programs	63. Self-medicating	19. Bridging the healthcare gap upon release	20. Increased prevalence of healthcare while incarcerated	33. Attitude
7. Supportive, understanding, and patient communities	73. Feeling that there is an inability to ask for help because of the “formerly incarcerated” label	21. Lack of education can contribute to recidivism	29. Barriers to getting medication while incarcerated (MOUD)	40. Broad legalization of drugs
13. Safe and secure housing	75. Starting to use drugs while in prison	35. Prison conditions contribute to the likelihood someone will use to cope with that trauma	38. Resources provided by some transitional housing programs	50. Easier to go to people that can relate to you
17. Providing people with a safe haven while incarcerated	76. Continuing to use while incarcerated	37. Freedom to choose to access mental health services	39. Accessing treatment through friends (e.g., being referred to treatment through trusted friends)	51. If you get turned down enough, you stop asking for help
18. Mental health counseling	77. Lack of knowledge surrounding using drugs	42. Greater access to alternative treatments	68. Increase access to treatment without consequences (e.g., increasing access to treatment without feeling stigmatized or singled out, making someone vulnerable to humiliation from other inmates)	52. Treated differently after being incarcerated
22. Lack of trauma-informed doctors	79. Lower tolerance when reentering society	44. Greater access to mental health services while incarcerated		53. After release, uncomfortable in your own skin
24. No access to Narcan while incarcerated, or when released		48. Willingness to access your support system		54. Stigma surrounding incarceration background that prevents people from getting and seeking help
25. Individual environment, such as being the subject of assault or violence, while incarcerated		49. People don’t know who to reach out to because of the lack of resources		58. Traumatic experiences from family members
28. Lack of continuity in care from incarceration to release		55. Turned down by drug treatment programs		59. Shame, being embarrassed to say no
30. Interruption in use during incarceration				60. Substances are readily available
34. Need to treat underlying trauma while incarcerated				61. Validation from friends and peers when using
36. Reaching out to people that you feel may need help				62. Peer pressure
43. Eliminating labels surrounding seeking help while incarcerated				64. Wanting to fit in
45. Eliminating the social stigma surrounding incarceration				65. Social environment
46. More support post-release from communities				67. Don’t know how to ask for help
47. Options for upward mobility in employment				69. Feeling the need to keep up the appearance of being okay even when you are not
57. Not used to encountering derivatives after release				70. Feeling like you can’t ask for help, so an “I don’t care” attitude develops
66. Unable to ask parole officers for help for fear of repercussions				71. Isolation
				72. Potential consequences for seeking help
				74. Mental health
				78. Psychological and acceptance factors regarding reentering society after being incarcerated
				80. Inability to see the world outside of your community
				81. Type of environment raised in during childhood
				82. Insecurity
				83. Self-worth

## Results

Eight individuals (ages 33–53) participated. Of the participants, six identified as male, one as female, and one as gender non-conforming. Four participants self-identified their race as Black/African American, three as White, and one as another race (i.e., not listed). For ethnicity, 3 participants identified as Hispanic/Latino, and 5 identified as not Hispanic/Latino. Participants resided in California, Colorado, North Carolina, and Virginia. Seventy-seven responses were produced by the eight participants from the initial prompt, “*What do you think are some of the main things that make people who have been in prison or jail more likely and less likely to overdose?*” After we combined identical responses and expanded responses that we considered to encapsulate more than one idea, there were a total of 83 factors (Table 1).

### Factors that influence overdose and substance use

**Fig. 1:**
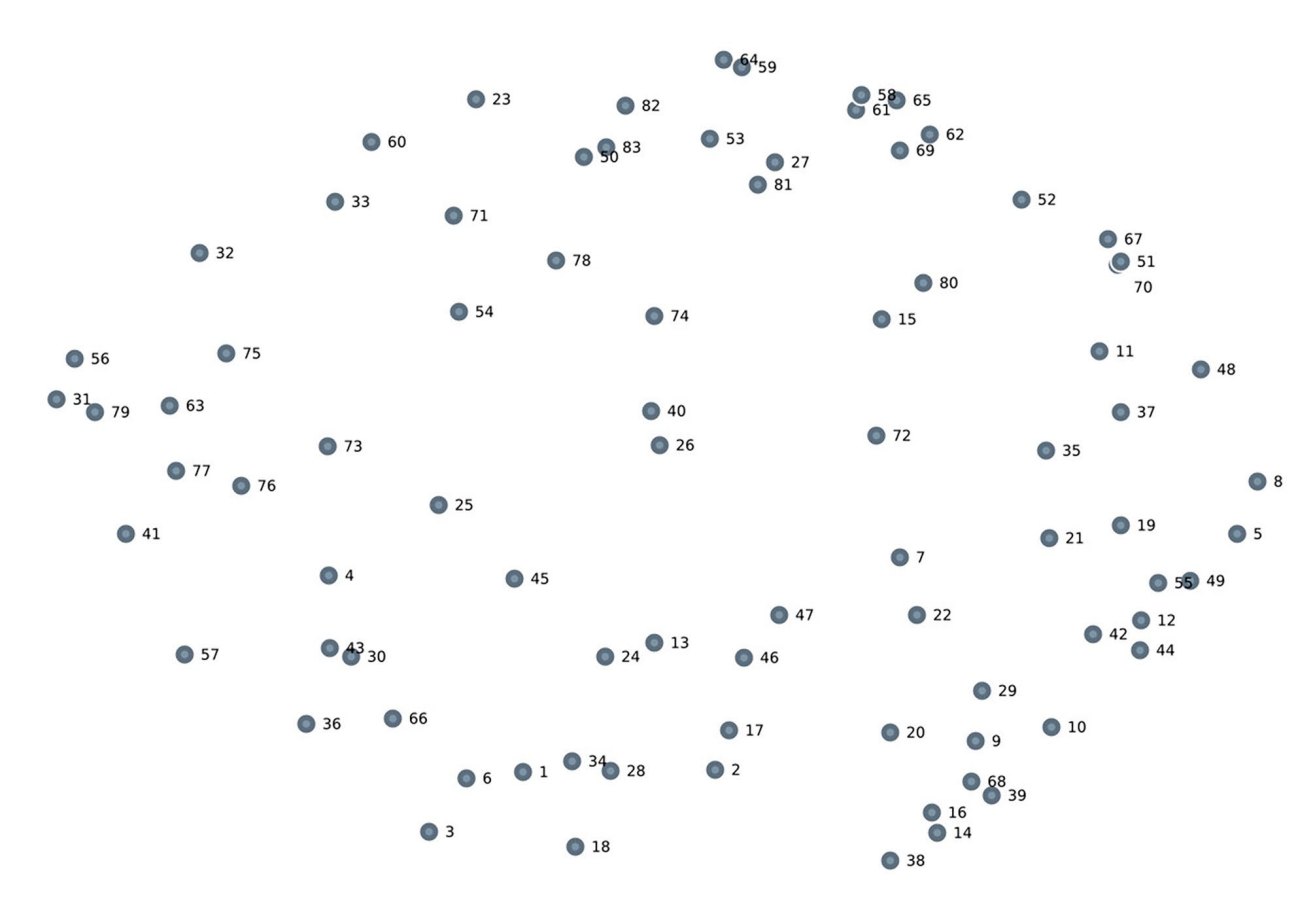
Point map created by multidimensional scaling algorithm. Each point represents an individual factor from the brainstorming session. Note: the corresponding factors to the numbers are outlined in detail in Table 1

Among the 83 factors (Fig. 1), the predominant factors influencing overdose and substance use that emerged from the brainstorming session were (1) negative attitudes towards oneself and society post-release, including low self-worth and feeling as if there is nowhere to turn for help, (2) lower drug tolerance after being incarcerated, (3) lack of access to treatment, and (4) lack of education and resources. Some individuals focused the discussion on the lack of services, lack of MOUD availability and accessibility, and the absence of post-release support systems compared to other participants.

### Clustering of factors that influence overdose and substance use

**Fig. 2:**
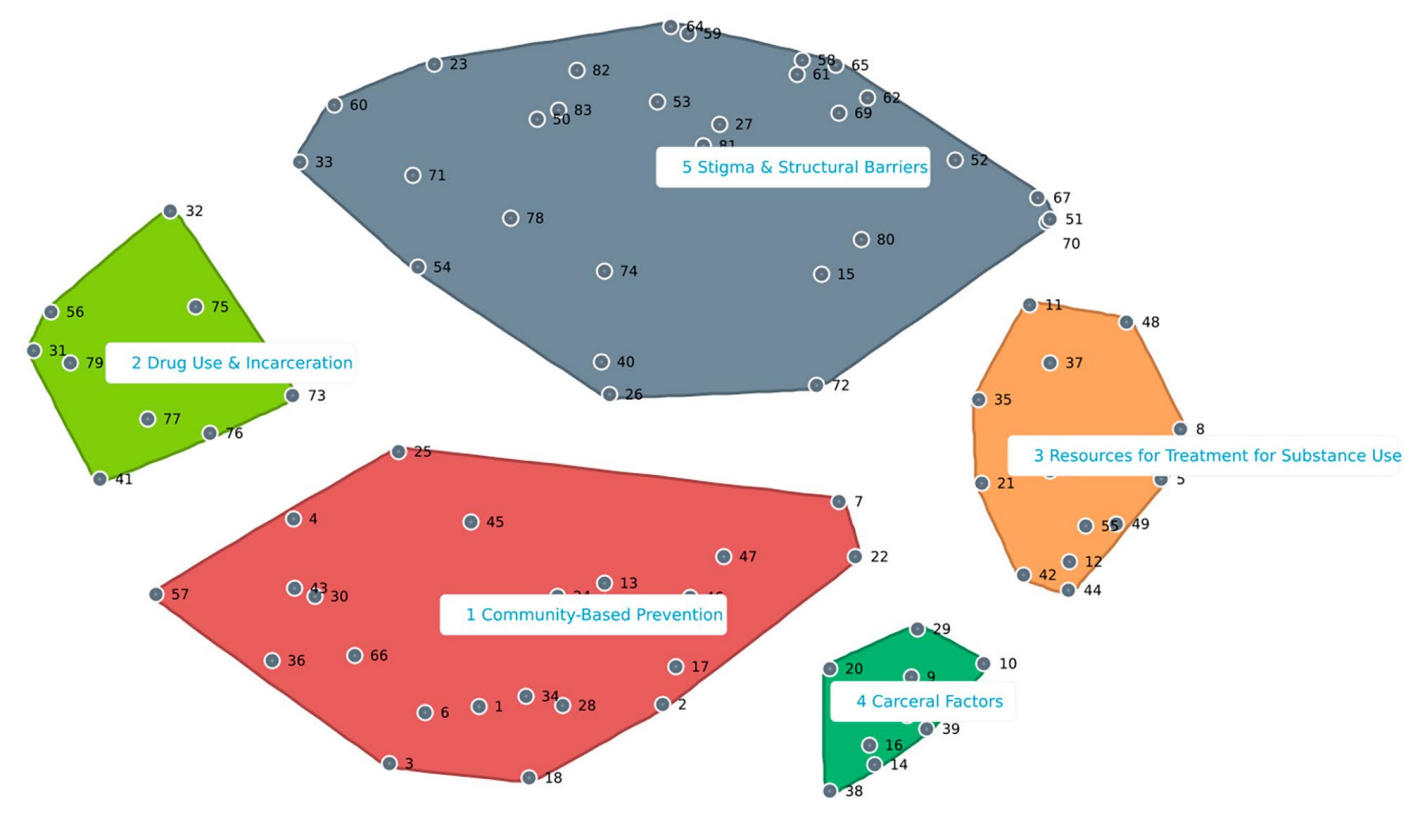
Five cluster map of factors associated with overdose following incarceration. Each factor is numbered within its respective cluster. 5 clusters were created from the point map as seen in Fig. 1

Aggregated sorting produced clusters within 11 different spatial maps all with a stress value of 0.34. The stress value was the same for all generated maps because they arose from the same point map. Due to a large number of maps, the study team chose 3 maps, a 9-cluster, an 8-cluster, and a 5-cluster solution, that they believed encompassed the overarching ideas and beliefs of participants based on the discussions and the list of factors. Study participants decided that the 5-cluster solution was the most representative spatial map (Fig. 2). The other cluster maps are included in the Supplemental Appendix (eFigs. 1 and 2).

Through an iterative process, the five clusters were labeled as follows: (1) *Community-Based Prevention*, (2) *Drug Use and Incarceration*, (3) *Resources for Treatment for Substance Use*, (4) *Carceral Factors*, and (5) *Stigma and Structural Barriers*. Each cluster related to a main issue that participants viewed to influence overdose and substance use, either positively or negatively. The specific factors within each cluster are noted in Table 1. *Community-Based Prevention* includes concepts around peer support post-release and educational and career opportunities. The *Drug Use and Incarceration* cluster contains notions about the unknown state of the drug supply post-release, continuing to use drugs while incarcerated, and having a lower tolerance when reentering society. *Resources for Treatment* include topics such as harm reduction, peer support, greater access to mental health services, and alternative types of treatment for substance use post-release. The *Carceral Factors* cluster consists of points concerning barriers to accessing treatment while incarcerated, and coordinating with organizations to ensure that people will have the necessary resources post-release, such as housing and trauma-informed medical treatment. Finally, the *Stigma and Structural Barriers* cluster include ideas encompassing prejudices against individuals who are previously incarcerated, personal attitudes post-release, and the social environment that exists upon reentry.

In the 5-cluster solution, *Stigma and Structural Barriers* is the largest cluster and geographically close to the other four clusters. This suggests that the stigma associated with past incarceration and the stigma surrounding overdose and substance use are closely interconnected with the other topics discussed. The *Community-Based Prevention* cluster is the second largest, and it neighbors *Stigma and Structural Barriers*, *Drug Use and Incarceration*, and *Carceral Factors*. It encompasses several issues that participants perceived as able to be addressed by appropriate community-level interventions and education. Types of community education include harm reduction, overdose prevention, and how communities can be supportive of individuals post-release. *Drug Use and Incarceration* is furthest from the *Carceral Factors* and *Resources for Treatment for Substance Use* clusters. *Resources for Treatment for Substance Use* is closest to the *Carceral Factors* cluster. The relative location of this cluster demonstrates the participants’ perception of the association between the lack of resources in communities and the lack of resources in jails and prisons. Far from *Drug Use and Incarceration*, but close to *Community-Based Prevention* and *Resources for Treatment for Substance Use*, the *Carceral Factors* cluster represents the participant’s beliefs that resources that should be provided in prisons and jails should also be carried over into communities after release. The relatively large distance between *Drug Use and Incarceration* and the *Carceral Factors* clusters may have been due to the disparate sorting completed by participants. For instance, participants sorted factors related to stigma in *Drug Use and Incarceration* and the *Stigma and Structural Barriers* clusters, and education-related topics in both *Community-Based Prevention* and *Resources for Treatment for Substance Use*.

**Fig. 3:**
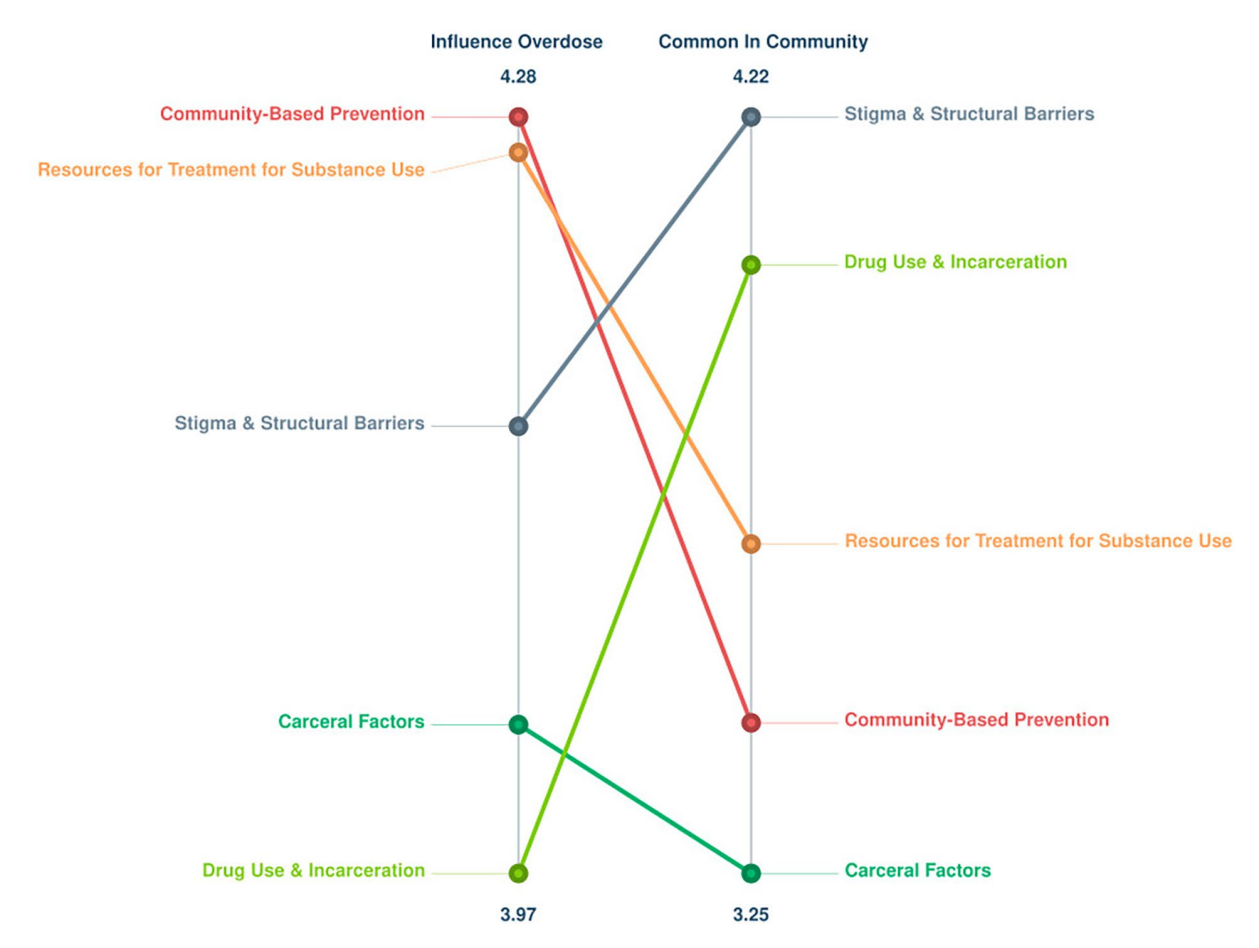
Pattern Match map depicting the relationships between clusters and their influence on overdose and prevalence in the community. On the left side, the rating for how much each cluster influences overdose is displayed, and on the right side is the rating for how common each cluster is in the community. Each cluster is connected to itself via a colored line. The clusters at the top of each side were rated as having the most impact

The ladder graph representation in Fig. 3 outlines the averaged ratings for each factor for the two rating prompts. The relative Pattern Match had a slightly negative correlation coefficient (*r* = -0.18), demonstrating an inverse relationship between the influence of a factor and its frequency in a community. For instance, the factors in the *Community-Based Prevention* cluster were perceived as highly influencing overdose, but they were rated as not being very common in the community. This reflects the participants’ beliefs that providing more community-based prevention methods could mitigate the issues revolving around substance use and overdose within the previously incarcerated population.

Regarding influencing overdose, the clusters listed from most important to least important are *Community-Based Prevention*, *Resources for Treatment for Substance Use*, *Stigma and Structural Barriers*, *Carceral Factors*, and *Drug Use and Incarceration*. When observing the importance of clusters concerning prevalence in the community, the order from most to least important is, *Stigma and Structural Barriers*, *Drug Use and Incarceration*, *Resources for Treatment for Substance Use*, *Community-Based Prevention*, and *Carceral Factors*.

**Fig. 4:**
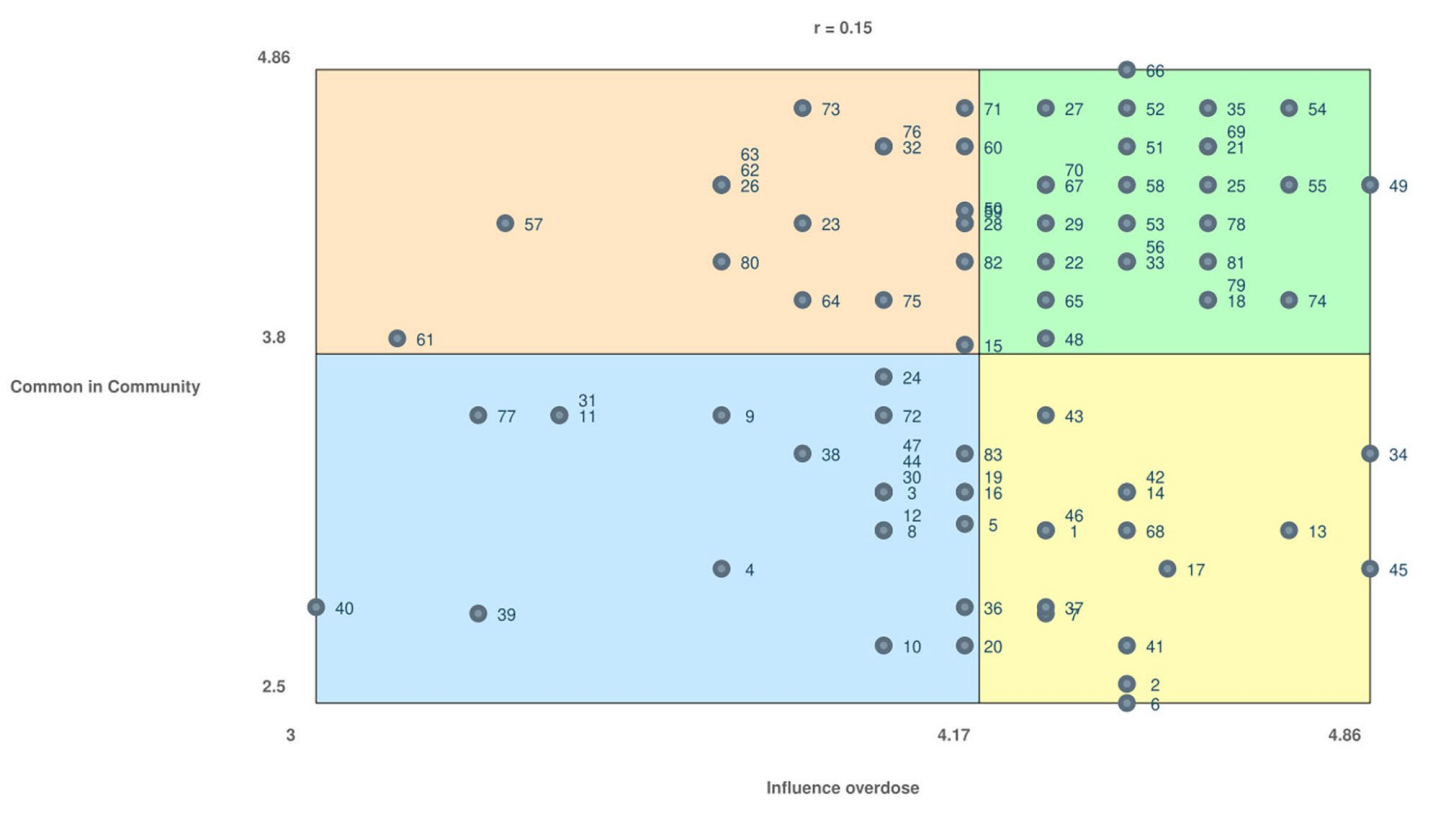
Go-Zone graphic representing the average rating values for each factor. The four quadrants are each related to how participants rated the individual factors, which can be viewed in Table 1. The orange quadrant are factors common in the community but has a low influence on overdose. The green quadrant are factors that are common in the community and has a high influence on overdose. The blue quadrant are factors that are not very common in the community, and have a low influence on overdose. The yellow quadrant are factors that are not very common in the community but have a high influence on overdose

Lastly, the Go-Zone chart resulted in a non-statistically significant correlation coefficient (*r* = 0.15), meaning that participants did not rate the individual factors similarly when compared to one another (Fig. 4). The orange quadrant represents high commonality in the community and a low influence on overdose (e.g., self-medicating, peer pressure, inability to see the world outside of your community). The green quadrant represents high commonality in the community and a high influence on overdose (e.g., feeling like you can’t ask for help, so an ‘I don’t care’ attitude develops, barriers to getting medication (MOUD) while incarcerated, fentanyl is in everything). The blue quadrant represents low commonality in the community and a low influence on overdose (e.g., accessing treatment through friends, harm reduction support for people that get released after-hours, opportunities to rise above the bare minimum). The yellow quadrant represents low commonality in the community and a high influence on overdose (e.g., eliminating labels surrounding seeking help while incarcerated, freedom to choose to access mental health services, trauma-informed staff). Each factor within each quadrant is in Table 1.

## Discussion

In this study, we used concept mapping as a tool for CEnR to identify factors at the intersection of substance use, overdose, and incarceration. A total of 83 factors were identified and from these factors, five clusters emerged: *Community-Based Intervention, Drug Use and Incarceration, Resources for Treatment for Substance Use, Carceral Factors, and Stigma and Structural Barriers*.

Participants identified several factors that may reduce overdose and substance use following release from incarceration, many of which were consistent with prior research. Factors that have been previously identified included barriers to getting MOUD while incarcerated, bridging the healthcare gap upon release, and arrests can play a role in self-esteem, leading to a higher likelihood of using drugs (Gideon, 2013; Grella et al., 2020; Zhang et al., 2022). Even though these are not new findings, their importance to overdose prevention is magnified given that they were directly identified by PWLE. This is particularly important as clinicians and policymakers are attempting to curb overdose deaths in the context of fentanyl. Ensuring that PWLE have their voices heard allows for culturally competent interventions to be developed that could substantially reduce overdose risk. Such interventions could include increasing access to basic needs, being surrounded by a supportive and understanding community, having access to harm reduction services, and addressing the stigma surrounding those who have had experience in the carceral system. Increasing access to basic needs such as housing, employment, and medical care, as well as being surrounding by a supportive and understanding community or having a relationship with a peer, have been shown in several studies to increase linkage to care, mobilize communities to advocate for systemic change, and prevent disease to create healthier populations (*A Compendium of Proven Community-Based Prevention Programs*, 2013; Chutuape et al., 2010; McLeod et al., 2020). Additionally, providing access to harm reduction programs, peers, and supplies (i.e., Narcan, testing strips), can decrease negative health outcomes such as overdose and disease transmission, bridge the gap for other essential services such as housing and health care, and can be a patient-centered and individualized approach to increase linkage to health care and decrease recidivism (Ashford et al., 2018; Barocas et al., 2015; Hunter et al., 2016; Hyde et al., 2022; Khan et al., 2022).

While each of the five clusters contain insights into intervenable components of the current correctional system, the Carceral Factors cluster contains factors that are immediately intervenable and evidence-based. Namely, the need to include MOUD to individuals during incarceration is a modifiable factor worth discussion. Several studies have noted that providing individuals access to MOUD while incarcerated increases community treatment engagement, reduces injection drug use and opioid use, and decreases fatal overdoses post-release (Cates & Brown, 2023; Klemperer et al., 2023; Martin et al., 2022). As described by a statewide analysis of the Rhode Island Department of Corrections, only 8% of their 1,600 participants who received MOUD suffered a fatal overdose post-release, compared to earlier studies citing a range of 16–26% fatal overdose post-release for those who did not obtain MOUD (Martin et al., 2022). Additionally, ensuring that harm reduction (e.g., naloxone and sterile injection equipment) is available during incarceration and post-release is critical. Incarceration causes disruptions in social networks and access to care, ultimately leading to the potential for someone to overdose post-release (Brinkley-Rubinstein et al., 2017; Joudrey et al., 2019; Ohringer et al., 2020). Guaranteeing access to MOUD, naloxone, and syringe services during incarceration, during the transition out of the carceral system, and post-release could reduce overdose risk and may also increase continuity of care (Brinkley-Rubinstein et al., 2017; Joudrey et al., 2019; Ohringer et al., 2020).

The issue of stigma was identified in each cluster by every participant and served as a foundational component of the concept map. While it has been well documented that stigma can decrease the likelihood of engaging in health care, participants emphasized that stigma may increase the risk of overdose following incarceration (Joudrey et al., 2019; Madden et al., 2021; Muncan et al., 2020; Nyblade et al., 2019). Stigma, both internalized and enacted, has been shown to increase the risk of overdose because of its potential effects on mental health and safe injection practices (Latkin et al., 2019). Additionally, stigma is associated with underfunding for substance use treatment, lack of enrollment for substance use treatment, and discrimination toward people who use drugs, particularly by law enforcement (Cheetham et al., 2022; Fadanelli et al., 2020; Wakeman & Rich, 2018). Unfortunately, despite its clear importance, stigma is not a variable that is routinely collected well in administrative datasets. Our study highlights a need to rethink which data are being collected, and how, by agencies that could potentially inform future research or the efficacy of programs and interventions (Brinkley-Rubinstein et al., 2019; Kaplowitz et al., 2022; Swartz et al., 2022).

CEnR is also integral to the development of public health interventions because it involves collaboration and partnership between researchers and community members. Concept mapping enhances the CEnR process by visually organizing the complex ideas, relationships, and patterns gleaned from community-driven discussion and research, which allows for a more comprehensive understanding of a community’s needs, strengths, and resources. Furthermore, concept mapping can serve as a tool to enhance the dissemination and uptake of research findings by making them more accessible to stakeholders and facilitating their translation into actionable steps. Following this project, several participants have remained involved with other research projects. The concept map was a first step in transforming the research team to incorporate the voices of those who are directly impacted by incarceration, substance use, overdose, and policies that target these issues. The collaboration with the participants established concrete results that can be used to push for systemic change starting at the community and local levels.

The insights gained from this study came directly from involving PWLE in the research process, which can further enrich policy discussions and inform interventions to be more relevant, targeted, and sustainable. Integration of PWLE should be included in policy discussions surrounding criminal legal penalties and substance use treatment options, as well as supportive programs like harm reduction, peer support, and transitional housing. Participants expressed that for interventions to be successful, their living and lived experience and expertise must be respected and integrated into stakeholder discourse. In doing so, researchers can foreground the concerns and challenges experienced by these communities more directly into the community program planning and policymaking processes. In particular, involving PWLE in the carceral system can dispel myths and help eliminate stereotypes about this population. Several participants noted that being provided with opportunities to be their “best selves” and share their experiences can help change systems that have historically and traditionally failed them.

### Limitations

The current project had limitations to note. First, the group of participants involved in this study is not representative of all types of incarceration, overdose experiences, and substance use experiences. Although our group of participants was small and not generalizable to the larger population of PWLE with the carceral system and substance use, the goal of our project was to gain a comprehensive understanding of the living and lived experiences of the participants. Additionally, the small group dynamic allowed for extensive collaboration and interactions between participants, which would have been more difficult in a larger group. As the participants were recruited from two different CABs, they may have had different experiences and expertise compared to other community members in other states where criminal legal policies and practices differ. Second, the cohort was limited to between seven and ten individuals, as recommended by expert opinion, to analyze experiences in depth, and encourage participants to have the opportunities to share as much as they were comfortable considering the sensitive nature of the research question. Third, the sorting activity proved to be the most difficult, as there were some factors in different clusters that should have seemingly been sorted together, such as variables associated with stigma, education, and basic necessities. However, the disparate piles created by participants may reflect their unique experiences. Fourth, the *groupwisdom* software does not calculate the correlation coefficients between individual clusters, which could have provided additional insight into the interrelatedness between factors and the common themes. Fifth, our stress value for the point map was high, but it was still in a reasonable range. One analysis found that the average stress value among 33 concept mapping projects ranged from 0.16 to 0.35 (Brennan et al., 2012). Having a high stress value may be indicative of a lack of agreement between the participants’ sorting data by the *groupwisdom* software.

## Conclusions

Our study provides an example of how concept mapping can be used to elicit a deep understanding of the relationships at the intersections of incarceration, overdose, and substance use using a community-engaged approach and be foundational for creating longstanding relationships. It contributes to the understanding that community engagement is necessary in order to overcome the barriers between research and lived experiences. Our concept map is a starting point for future community-engaged research with this population that expands on the understanding surrounding the special care and consideration that marginalized communities need. We found that stigma is a strong theme prevalent throughout all clusters, participants feel that they are not provided with adequate support while incarcerated or post-release, and there is a strong desire for peer support. Concept mapping in conjunction with the community members’ perceptions, beliefs, and living and lived experiences, provides the research community with insights that would otherwise be unnoticed. Structural factors of drug use and incarceration have been intensely researched, but the intersection between the two needs additional work and the incorporation of people with lived experience, and concept mapping provides a tool with which to do this. The integration of PWLE into research, public health, and policy is essential for addressing the issues that continue to trouble this vulnerable population.

### Electronic supplementary material

Below is the link to the electronic supplementary material.

Supplementary Material 1

## Data Availability

The data that support the findings of this study are available on request from the corresponding author, J.A.B. The data are not publicly available due to their containing information that could compromise the privacy of research participants.
