# Supplementary material for: Identifying structural risk factors for overdose following incarceration: a concept mapping study
Source: Health Justice. 2024 Mar 12;12:11. doi: 10.1186/s40352-024-00265-w (PMC10936003; doi:10.1186/s40352-024-00265-w)
Supplement: Supplementary file 1 — Supplementary Material 1 [file 40352_2024_265_MOESM1_ESM.docx]

**Supplemental Online Content**

**Title:** Identifying Structural Risk Factors for Overdose Following Incarceration: A Concept Mapping Study

**Authors:** Samantha K. Nall^1^, Cole Jurecka^1^, Anthony Ammons Jr.^2,3^, Avel Rodriguez^4^, Betsy Craft^4,5^, Craig Waleed^3,6^, Daniel Dias^4,7^, Jessie Henderson^1,4^, Joshua Boyer^3,8^, Kristina Yamkovoy^1^, Pallavi Aytha Swathi^1^, Prasad Patil^9^, Forrest Behne^11^, Katherine LeMasters^10^, Lauren Brinkley-Rubinstein^11^, Joshua A. Barocas^1*^

***Corresponding author:**

Joshua A. Barocas, MD

University of Colorado School of Medicine

8th Floor, Academic Office 1

Mailstop B180

12631 E 17th Ave

Aurora, CO 80045

+1-314-348-3278

[Joshua.Barocas@CUAnschutz.edu](mailto:Joshua.Barocas@CUAnschutz.edu)

**Supplement 1**. eAppendix

**Table of Contents**

Concept Mapping Syllabus……………………………………………………………3-6

Alternative Cluster Map Solutions……………………………………………………7-8

**List of Contents**

eTable 1. Concept Mapping Schedule: In Detail………………………………………5-6

eFigure1. 9-cluster solution……………………………………………………………7

eFigure2. 8-cluster solution……………………………………………………………8

**Concept Mapping Syllabus**

Community Participatory Action Research

Identifying structural risk factors for overdose following incarceration

The Missing US Project

**Principal Investigator**

Josh Barocas, MD

Associate Professor of Medicine

Divisions of Internal Medicine and Infectious Diseases

University of Colorado School of Medicine

Joshua.Barocas@Cuanschutz.edu

**Co-Investigators/Collaborators**

Lauren Brinkley-Rubinstein, PhD

Prasad Patil, PhD

Tina Yamkovoy, MPH

Samantha Nall, MPH

Cole Jurecka, MPH

Katherine LeMasters, PhD

Forrest Behne, MPHc

**Objective**

The aims of this series are (1) to apply a participatory action research approach to identify the factors that people with lived experience perceive to be influencing overdose following incarceration, (2) map the relationships and interactions between these factors, in order to elucidate theories of overdose risk, and (3) incorporate these community-derived factors into mathematical prediction models to help develop tailored overdose prevention programs for people leaving the carceral system.

**Collaboration, participation, and partnership**

This collaboration is completely voluntary, and participants will be compensated for their participation. Community members willing to participate will be consented using an informed consent process. We offer co-authorship to any participant who wants to be included in any manuscripts resulting from this process. This collaboration will work best if there is consistent participation throughout all sessions, however, we understand if people are unable to attend every session. We will try to provide meeting notes to participants unable to attend a session.

**eTable 1.** Concept Mapping Schedule: In Detail

| **Session** | **Dates** | **Activities** |
| --- | --- | --- |
| ***Introduction*** | ***August/September*** | ***Introduction & Community Preparation*** |
| In this first session, we will introduce community members to the project, which includes using big data and mathematical models to predict overdose following incarceration. We will explore what community voices add to this project and discuss how we can use concept mapping to incorporate voices into mathematical models. Concept mapping is a structured method for translating complex qualitative data into a pictorial form and the goal is to capture shared and unique views of individuals in a community. We do concept mapping because purely epidemiological approaches fail to capture the complex dynamics that influence health and because community members often hold unique insider perspectives. The overall goal of this session is to help community members become familiar with the team, understand the “big ideas” relevant to this project, and begin to build trusting relationships. | | |
| ***Session 1*** | ***Late September*** | ***Community Brainstorming*** |
| The goal of this session will be to convene willing (and consented) community members to begin the concept mapping process. In this meeting, participants will be asked to respond to the following probe: “What do you think are some of the main things that make people who have been in prison or jail more likely to overdose? What are the main things that make people who have been in prison or jail less likely to overdose?” There will be follow-up probes to encourage discussion. Participants will be encouraged to include both positive and negative factors – that is, factors that cause or reinforce overdose as well as factors that reduce overdose. After the first meeting the researchers will combine the lists of factors produced by the group by removing duplications and integrating similar factors. | | |
| ***Session 2*** | ***Mid-October*** | ***Community Sorting & Rating*** |
| The goal of this session will be to perform the next step in the concept mapping process. Participants will review the list of generated factors (from the last meeting) to confirm that this accurately and comprehensively represents their perceptions, ensuring that principles of communal self-determination and collaborative partnership are upheld. The individual participants will then be asked to rate each factor on a 5-point likert scale in terms of 1) how much each factor increases overdose risk, and 2) how common the factor is in the community. Thereafter, each participant will be given a pile of cards, with each factor printed on a separate card. We will attempt to use electronic software to accomplish this. Using the instruction, “sort the cards in a way that answers the question ‘what are the factors that influence overdose in your community’ and give each pile of cards a name”, participants will be asked to sort the factors into categories. The research team will explain this task using a familiar metaphor of sorting laundry. Participants will be asked to sort the factors into more than two piles and less than 30 piles. | | |
| ***Session 3*** | ***November*** | ***Community Interpretation*** |
| Between sessions 2 and 3, researchers will use concept mapping software (Concept Systems) to analyze responses from sorting and rating. During this third session, the group will discuss how the computer program used the information that the participants initially brainstormed, ranked and sorted to display the factors as points on a map. Graphs and maps will be used to generate group conversations regarding the issues that influence overdose in the community. These discussions will be recorded and later transcribed. These results will be fed back to the community as a launchpad for future work in reducing overdose following incarceration. | | |

**Alternative Cluster Map Solutions**


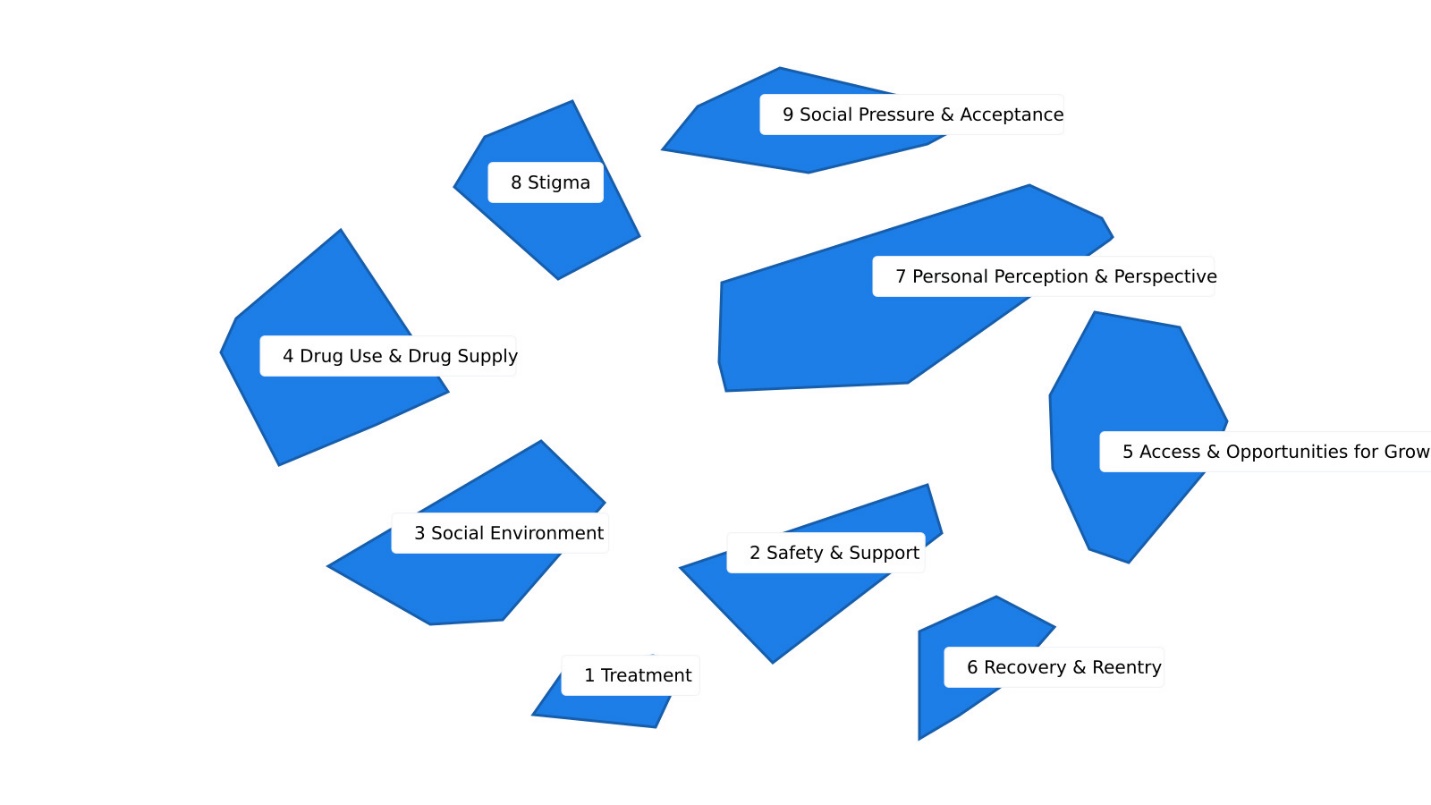


**eFigure 1.** 9-cluster solution.


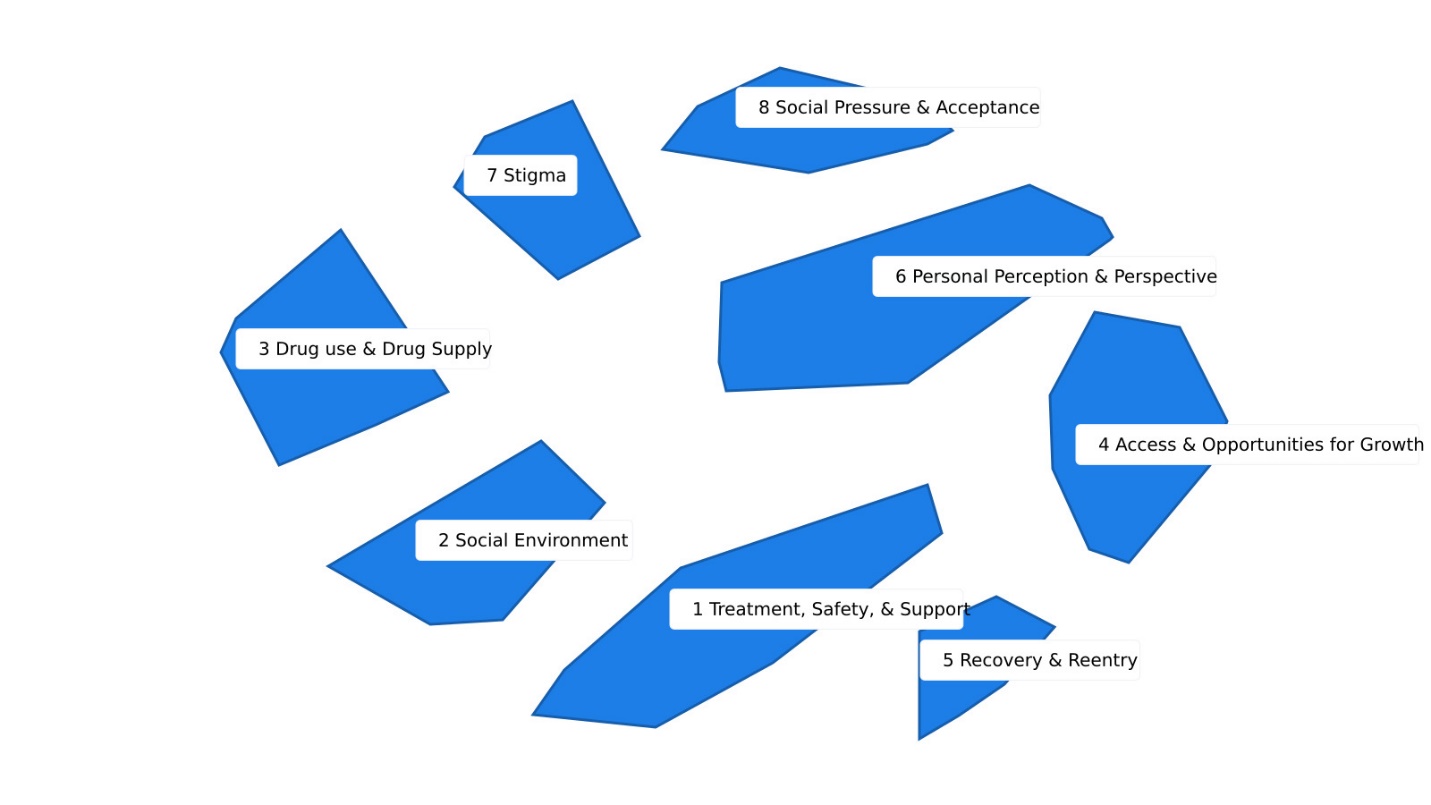


**eFigure 2**. 8-cluster solution.
